# Mixed material wear particle isolation from periprosthetic tissue surrounding total joint replacements

**DOI:** 10.1002/jbm.b.35076

**Published:** 2022-05-09

**Authors:** Ashley A. Stratton‐Powell, Sophie Williams, Joanne L. Tipper, Anthony C. Redmond, Claire L. Brockett

**Affiliations:** ^1^ Institute of Medical and Biological Engineering, School of Mechanical Engineering University of Leeds Leeds UK; ^2^ School of Biomedical Engineering University of Technology Sydney Ultimo New South Wales Australia; ^3^ NIHR Leeds Biomedical Research Centre Leeds Teaching Hospitals NHS Trust Leeds UK; ^4^ Leeds Institute for Rheumatic and Musculoskeletal Medicine, School of Medicine University of Leeds Leeds UK

**Keywords:** foreign body reactions (response), implant retrieval, total joint replacement, tribology, wear debris

## Abstract

Submicron‐sized wear particles are generally accepted as a potential cause of aseptic loosening when produced in sufficient volumes. With the accelerating use of increasingly wear‐resistant biomaterials, identifying such particles and evaluating their biological response is becoming more challenging. Highly sensitive wear particle isolation methods have been developed but these methods cannot isolate the complete spectrum of particle types present in individual tissue samples. Two established techniques were modified to create one novel method to isolate both high‐ and low‐density materials from periprosthetic tissue samples. Ten total hip replacement and eight total knee replacement tissue samples were processed. All particle types were characterized using high resolution scanning electron microscopy. UHMWPE and a range of high‐density materials were isolated from all tissue samples, including: polymethylmethacrylate, zirconium dioxide, titanium alloy, cobalt chromium alloy and stainless steel. This feasibility study demonstrates the coexistence of mixed particle types in periprosthetic tissues and provides researchers with high‐resolution images of clinically relevant wear particles that could be used as a reference for future in vitro biological response studies.

## 1 INTRODUCTION

Aseptic loosening is the most common cause of long term total joint replacement (TJR) failure in the United Kingdom^1^ and is characterized by the loosening of a fixed component in the absence of infection. This phenomenon is often the result of a combination of biological and mechanical events leading to the destruction of the bone‐implant interface. The biological theory for the cause of aseptic loosening identifies wear debris as the most important factor.^2^, ^3^ Wear debris within a critical size range (~0.2–0.8 μm) has been shown to activate human macrophages and osteocytes which can result in a proinflammatory response, osteoclastogenisis and localized bone resorption or osteolysis.^4^, ^5^, ^6^ Despite aseptic loosening being the leading cause of TJR failure to date, this complication has declined in prevalence since the introduction of highly wear resistant biomaterials (e.g., highly crosslinked and anti‐oxidant polymers).^7^, ^8^, ^9^ With approximately 58% and 82% of hip and knee replacements enduring 25 years of use, respectively,^10^, ^11^ understanding the characteristics and effects of wear debris remains essential for determining the long term performance of devices.

The volume of wear produced at the primary bearing surface has decreased substantially over time and is suggested to be the main reason for reduced revision rates of hip replacements with up to 13 years of clinical follow‐up.^8^, ^9^ Despite this, factors other than wear volume, such as wear particle size, shape and chemistry, are known to affect the biological response associated with implants.^12^ How these other important factors change between devices, or over the lifetime of an individual device, is not yet understood.^13^

The progressive reduction of wear at the primary bearing surface has made isolating lower volumes of wear particles more challenging, requiring the development of more sensitive methods.^14^, ^15^, ^16^, ^17^ In addition, the focus of research has somewhat changed to surfaces other than the primary bearing, such as the backside of acetabular liners or cups and corrosion at modular junctions.^18^, ^19^, ^20^, ^21^ These changing requirements emphasize the importance of isolating and characterizing wear particles produced across all wear modes.^22^

The production of mixed wear particle populations was recognized in early generations of joint replacement^23^ and wear particle isolation methods were developed to isolate both high and low density materials concurrently.^24^, ^25^, ^26^, ^27^, ^28^ Since the development of these methods, several key principles have been established for isolating specific materials,^29^ all of which would need to be considered when multiple materials are being studied. For example, it is generally accepted that acid and base digestion results in a more complete protein digestion.^27^, ^30^ Yet, these aggressive digestion techniques dissolve certain wear particle types like polymethylmethacrylate (PMMA), calcium phosphate and nanoscale metal wear particles, rendering them unmeasurable.^25^, ^26^, ^31^, ^32^ Also, these methods did not have the sensitivity nor resolution to isolate and characterize nanoscale wear particles. Maloney, et al.^24^ were the first to use enzymatic digestion in a dual material isolation method. The average size of the isolated UHMWPE and titanium wear particles was 0.5 ± 0.3 μm and 0.7 ± 0.3 μm, respectively. These results have since been generally representative of the wear particle sizes isolated from tissue samples for both THR and TKR.^25^, ^26^, ^33^, ^34^ However, the size distribution was skewed in favor of the larger and visually defined particle types which were often agglomerates of wear debris rather than individual particles. Ultimately, these limitations led researchers to optimize methods for isolating specific materials, such as UHMWPE^33^ or cobalt chromium alloy associated with metal‐on‐metal devices^31^, ^35^ as opposed to broader multi‐material approaches.

Highly sensitive wear particle isolation methods have since been developed.^14^, ^15^, ^16^, ^17^, ^36^ These modern methods are effective at isolating, characterizing, and imaging wear particles of all size ranges from highly wear‐resistant devices. Yet, these methods remain focused on the highest wearing materials, often associated with the primary bearing surface (i.e. UHMWPE in a metal on UHMWPE construct) and do not attempt to capture the full range of high‐ and low‐density particle types present within individual human tissue samples.

In this research, a more exploratory approach to isolating wear particles was taken, whereby a broader spectrum of particle types was targeted rather than one specific material type. The utility of such an approach is for where the composition and morphology of wear affecting the surrounding biology is less obvious, unknown or where primary bearing wear is considered to be of less concern, such as in devices used for smaller joints (e.g., shoulder, elbow, ankle, finger, spine). A modified version of two established state of the art wear particle isolation methods was developed.^16^, ^36^ The aim of this study was to determine whether the modified method can reliably isolate and characterize UHMWPE (<1.0 g cm^−3^) and high‐density wear particles (>2.0 g cm^−3^) from human tissue samples. Periprosthetic tissue from THR and TKR patients at revision surgery containing generally well understood wear particle types were characterized and compared to the literature.

## 2 MATERIALS AND METHODS

Ethics approval for this research was obtained by an application to the UK Health Research Authority (ref: 09/H1307/60). Periprosthetic tissue samples were retrieved from 18 patients undergoing revision surgery for failed THR (*n* = 10) and TKR (*n* = 8). The average patient age at implantation was 64 years (range 48–79) for THR and 71 years (range 62–84) for TKR. The average implantation time for the THR and TKR devices was 142 months (range 9–288) and 167 months (range 86–224), respectively. Specific device brands were not known for some of the tissue samples but the articulation type, indications for surgery, fixation type, device materials, reason for revision and patient demographic details were recorded where possible (Table 1).

**TABLE 1:** Demographic and device material information associated with total hip replacement and total knee replacement tissue samples analyzed

	Side	Sex	Diagnosis	Age (years)	Reason for revision	Implant time (months)	Bearing type	Fixation		Device materials			
**THR**								**Cup**	**Stem**	**Cup**	**Liner**	**Head**	**Stem**
1	R	F	OA	79	Dislocation	NK	MoP	Cement	NK	UHMWPE^a^	n/a	CoCr	NK
2	R	F	OA	48	Stiff Joint	108	NK	NK	C/less	NK	NK	Ceramic	Ti Alloy
3	R	M	AVN	49	Pain	288	CoP	C/less	C/less	Ti Alloy	UHMWPE	Ceramic	Ti Alloy
4	R	M	NK	72	Liner Dissociation	71	CoP	C/less	C/less	CoCr	UHMWPE	Ceramic	Ti Alloy
5	R	M	OA	73	Pain, Aseptic Loosening	250	MoP	Cement	Cement	Ti Alloy	UHMWPE	CoCr	Ti Alloy
6	R	F	NK	58	Pain, Aseptic Loosening	168	CoP	Cement	NK	UHMWPE	n/a	Ceramic	NK
7	R	F	OA	78	Recurrent Dislocation	192	MoP	Cement	Cement	UHMWPE	n/a	SS	SS
8	NK	NK	NK	NK	NK	NK	MoP	Cement	Cement	UHMWPE	n/a	SS	SS
9	L	M	NK	53	Septic Loosening	52	CoP	Cement	Cement	UHMWPE	n/a	Ceramic	SS
10	R	M	OA	70	Aseptic Loosening	9	NK	NK	C/less	NK	NK	CoCr	Ti Alloy
**TKR**								**Fem**	**Tib**	**Fem**	**Insert**	**Tib**	
1	L	F	RA	62	Aseptic Loosening	182	MoP	Cement	Cement	CoCr	UHMWPE	Ti alloy	
2	R	F	OA	70	Tib Component Migration	101	MoP	Cement	Cement	CoCr	UHMWPE	Ti alloy	
3	L	M	OA	73	Aseptic Loosening	86	MoP	Cement	Cement	CoCr	UHMWPE	CoCr	
4	R	M	OA	84	Aseptic Loosening	181	MoP	Cement	Cement	CoCr	UHMWPE	Ti alloy	
5	R	F	RA	62	Instability, Wear	224	MoP	Cement	Cement	CoCr	UHMWPE	Ti alloy	
6	R	F	NK	73	NK	NK	MoP	Cement	Cement	CoCr	UHMWPE	Ti alloy^b^	
7	R	F	RA	63	Wear	219	MoP	Cement	Cement	CoCr	UHMWPE	Ti alloy	
8	L	F	RA	81	Aseptic Loosening	180	MoP	Cement	Cement	CoCr	UHMWPE	Ti alloy	

Abbreviations: THR, Total hip replacement; TKR; Total knee replacement; MoP, Metal‐on‐Polyethylene; CoP, Ceramic‐on‐Polyethylene; L, Left; R, Right; M, Male; F, Female; OA, Osteoarthritis; RA, Rheumatoid Arthritis; AVN, Avascular Necrosis; Fem, Femoral Component; Insert, Bearing Insert; Tib, Tibial Component; NK, Not Known; n/a, Not applicable; Cement, Cemented fixation; C/Less, Cementless fixation; UHMWPE, Ultra‐high molecular weight polyethylene; Ti alloy, Titanium alloy; CoCr, Cobalt chromium alloy; SS, Stainless steel.

^a^ Posterior Lip Augmentation Device (PLAD) made from stainless steel was also implanted.

^b^ Worn‐through tibial tray observed.

### 2.1 Wear particle isolation method

**FIGURE 1:**
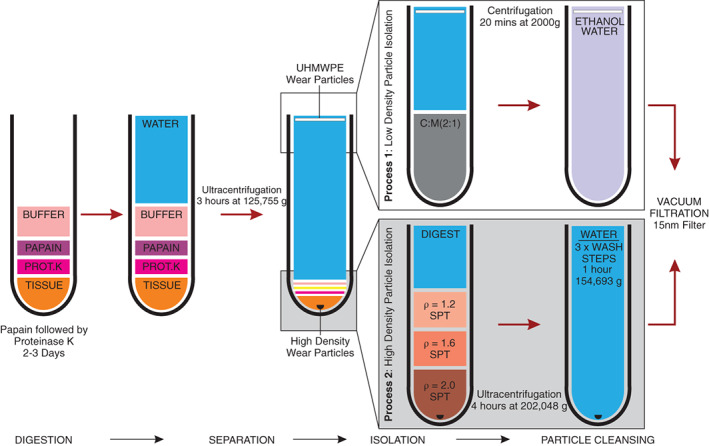
Modified wear particle isolation method. Prot.K, Proteinase K; SPT, Sodium Polytungstate; C:M (2:1); Chloroform: Methanol (2:1) mix; ρ, Density

A novel method was developed to separate high‐ and low‐density materials by density (Figure 1). The separation step used ultracentrifugation to float particles with a material density of less than ~1.0 g cm^−3^ (e.g., UHMWPE; Density = 0.93 g cm^−3^) and to sediment particles with a material density greater than ~2.0 g cm^−3^ (e.g. calcium phosphate; density = ~3.1 g cm^−3^). Low‐density particles were treated using an adapted version of the method published by Richards et al.^36^ (referred to as Process 1), whereas an adapted version of the method by Lal et al.^16^ was used to process high density particles (referred to as Process 2).

### 2.2 Sample preparation and digestion

Following retrieval, tissue samples were stored in formaldehyde (10% v/v) for at least 7 days followed by long‐term storage in ethanol (70% v/v) at room temperature. Approximately 1 gram of tissue was randomly dissected from each donor sample and cut into small pieces (~1 mm^3^). To preserve calcium phosphate and metallic particles, acid and base digestion methods were avoided. The tissue was added to a 38.5 ml ultracentrifuge tube (Beckman Coulter, USA) coated with siliconising fluid (Surfasil, Sigma UK) and digested over 2 days using 1 mg ml^−1^ papain and 0.1 M MOPS Buffer with 20 mM L‐Cysteine (pH 6.5) at 60°C for 24 h; then 1 mg ml^−1^ proteinase K in a mix with 0.5% (w/v) sodium dodecyl sulphate (SDS), HEPES Buffer (working concentration 0.1 M) (pH 7.8), 3 mM CaCl_2_ and ultrapure water for another 24 h at 37°C. The samples were agitated at 240 rpm throughout digestion. All reagents were filtered through a 20 nm Whatman® Anodisc membrane filters (GE Whatman, UK) to remove external contaminants prior to use.

### 2.3 Particle separation step

Ultra‐pure water was added to fill each ultracentrifugation tube and the tubes were centrifuged at 125,755 g at 20°C for 3 h (Optima L80 ultracentrifuge, Beckman Coulter, USA). All materials with a density greater than water (~1.0 g cm^−3^) which included: proteins, metal, calcium phosphate, bone cement, extracellular matrix, and high‐density lipids, were pelleted. The supernatant contained materials with a density less than water such as UHMWPE wear particles and lipids. The supernatant for each sample was decanted 5 ml at a time into clean glass universals ready for process 1. The pellet of particles remaining in the ultracentrifuge tube was continued to process 2.

### 2.4 Process 1: Isolation of particles with a density <1.0 g cm^−3^

Ten milliliters of chloroform: methanol (2:1 v/v) mix was added to each glass universal, agitated at 240 rpm for 2 min and incubated for at least 24 h at room temperature. The chloroform: methanol mix separated creating two phases. Lipids accumulate at the interface of the two phases, whereas salts precipitate and sink to the bottom. The glass universals were centrifuged at 2000*g* for 20 min at room temperature. The supernatant was removed with a glass pipette and added to another 10 ml of chloroform: methanol. The samples were shaken and incubated for at least 24 h at room temperature. The supernatant was removed with a glass pipette and accumulated into a 250 ml high speed centrifugation tube. Twenty milliliters of filtered ethanol were added, followed by 60 ml of ultrapure water. Finally, the sample was centrifuged for 2 h at 10,000*g* at 4°C, after which, the sample was ready for filtration and characterization.

### 2.5 Process 2: Isolation of particles with a density >2.0 g cm^−3^

A density gradient was compiled in a 12.5 ml ultracentrifuge tube with 2 ml of 60% (v/v) (*ρ* = 2.0 g cm^−3^), 2 ml of 40% (v/v) (*ρ* = 1.6 g cm^−3^) and 2 ml of 20% (v/v) (*ρ* = 1.2 g cm^−3^) concentrations of sodium polytungstate, each carefully layered on top of each other. The pelleted sample from the separation step was sonicated with 2 ml of ultrapure water and carefully pipetted on top of the density gradient. Samples were centrifuged for 4 h at 202,048 g at 25°C. The sodium polytungstate was discarded and the pellet was sonicated with 2 ml of ultrapure water. The sample was transferred to a clean 12.5 ml ultracentrifuge tube and washed in ultrapure water three times followed by centrifugation at 154,693*g* at 20°C for 1 h. In between each centrifugation wash, the ultrapure water was exchanged. Finally, the pellet of wear particles, which was free from contaminants, was filtered and characterized.

### 2.6 Particle characterization

High‐ and low‐density particles were filtered onto separate 0.015 μm filter membranes (GE Whatman, UK), left to dry overnight at room temperature and mounted directly onto aluminium stubs prior to being coated with carbon to a thickness of 5 nm. The filter membranes were imaged using a Hitachi SU8230 high resolution cold‐field emission scanning electron microscope (CFE‐SEM) at between 50‐ and 200,000‐times magnification. The backscatter detector on the SEM was used to highlight high‐density materials.^37^ Particle composition was identified using energy‐dispersive X‐ray (EDX) spectroscopy. Particle morphology was described qualitatively in accordance with ASTM F1877‐16.^38^ Quantitative metrics such as major diameter (*D*_max_), aspect ratio and roundness measurements were also captured for each particle manually using ImageJ.^39^

### 2.7 Statistics

Non‐parametric descriptive statistics (e.g., median size, interquartile range) were reported for wear particle size and parametric characteristics were reported for morphology (e.g., mean aspect ratio with standard deviation). The proportion of wear particles in the submicron (<1 μm), 1–10 μm and >10 μm size ranges were also reported. Inferential statistics were not undertaken because the specific devices and materials from which the particles originated could not be identified with certainty and could not be compared appropriately. All analyses were performed using SPSS Statistics Version 22 (IBM Corp, USA).

## 3 RESULTS

High‐ and low‐density wear particles were isolated and characterized from all THR and TKR tissue samples. At least three different material types were isolated from 6 of 10 THR samples and 5 of 8 TKR samples (Table 2). UHMWPE was identified by its morphology and was assumed, following the exclusion of contaminants, to be the only low‐density material isolated in process 1 (low‐density particles). Contaminants such as silica and residual proteins were identified at low frequencies and excluded with the aid of EDX analysis. Following process 2 (high density particles), six different material types were identified. The most common materials were those conventionally used as the matrix and additives in bone cement, which included: polymethylmethacrylate (PMMA), zirconium dioxide (ZrO_2_) and barium sulphate (BaSO_4_). Three conventional metals commonly used in joint replacement implants and accessories were also identified: titanium alloy (Ti Alloy), stainless steel (SS) and cobalt chromium alloy (CoCr). The composition of the isolated particles corresponded well with the fixation of the explanted devices and the UHMWPE components (Table 2). However, the wear particle types consistent with the metallic components, such as the head/stem of THRs and femoral/tibial components of TKRs, were notably less common. Neither of the two confirmed cementless constructs (THR #3 and #4) featured wear particles associated with bone cement. All TKR devices were cemented, and bone cement particles were identified in all except one device (TKR #7). Bone cement‐related particles were not identified in one completely cemented THR (THR #5).

**TABLE 2:** Number and type of isolated wear particles with a comparison between the isolated particle materials and the known explant materials

	Total	UHMWPE	PMMA	ZrO_2_		BaSO_4_	Ti Alloy	SS	CoCr	Discrepancies between the isolated particle materials and the known explant materials
				Mulberries	Granules					
THR										
1	468	340	0	0	0	100	0	28	0	Head material not identified
2	772	431	0	71	270	0	0	0	0	Stem and head materials not identified
3	465	274	0	0	0	0	191	0	0	All materials identified
4	306	222	0	0	0	0	84	0	0	Head material not identified
5	213	195	0	0	0	0	2	14	2	Fixation materials not identified. Origin of SS unknown
6	514	224	0	26	264	0	0	0	0	Stem and head materials not identified
7	214	175	0	1	19	0	0	19	0	All materials identified
8	360	120	34	3	203	0	0	0	0	Stem and head materials not identified
9	182	163	0	2	0	0	0	17	0	All materials identified
10	321	135	11	13	162	0	0	0	0	Stem and head materials not identified
All	3815	2279	45	116	918	100	277	78	2	
TKR										
1	240	204	0	0	0	32	0	4	0	Fem/Tib materials not identified. Origin of SS unknown
2	319	78	10	25	205	0	0	0	1	Tib materials not identified
3	225	174	0	0	3	0	0	39	9	Origin of SS unknown
4	500	289	0	0	1	0	210	0	0	Fem materials not identified
5	446	175	0	10	261	0	0	0	0	Fem/Tib materials not identified
6	461	249	0	0	48	0	164	0	0	Fem materials not identified
7	409	219	0	0	0	0	190	0	0	Fem and fixation materials not identified
8	355	303	0	0	52	0	0	0	0	Fem/Tib materials not identified
All	2955	1691	10	35	570	32	564	43	10	

Abbreviations: THR, Total hip replacement; TKR; Total knee replacement; UHMWPE, ultra‐high molecular weight polyethylene; PMMA, polymethylmethacrylate; ZrO_2_, zirconium dioxide; BaSO_4_, barium sulphate; Ti alloy, titanium alloy; SS, stainless steel; CoCr, cobalt chromium alloy; Fem, femoral component; Tib, tibial component; PLAD, posterior lip augmentation device.

**FIGURE 2:**
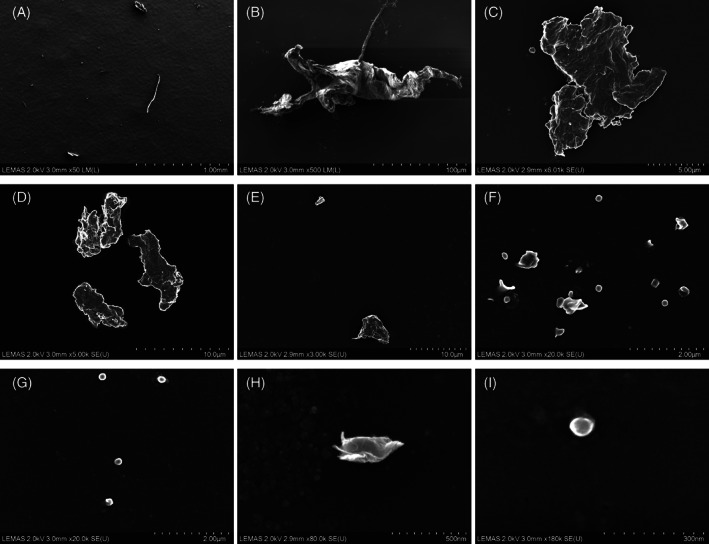
Examples of UHMWPE particles from a range of sizes. Organized from largest (A) to smallest (I)

The mean number of UHMWPE particles characterized for THR and TKR tissue samples was 228 (range 120–431) and 211 (range 78–303), respectively. UHMWPE particles were observed as fibrils, flakes, and granules for both joint replacement types (Figure 2). For both THR and TKR, 73% of the isolated UHMWPE particles were in the submicron size range, which was the smallest particle population identified overall. However, the largest individual wear particle was also UHMWPE with a major diameter of 465 μm. This particle was isolated from a THR sample and was fibrillar with straight morphology (Figure 2A).

**FIGURE 3:**
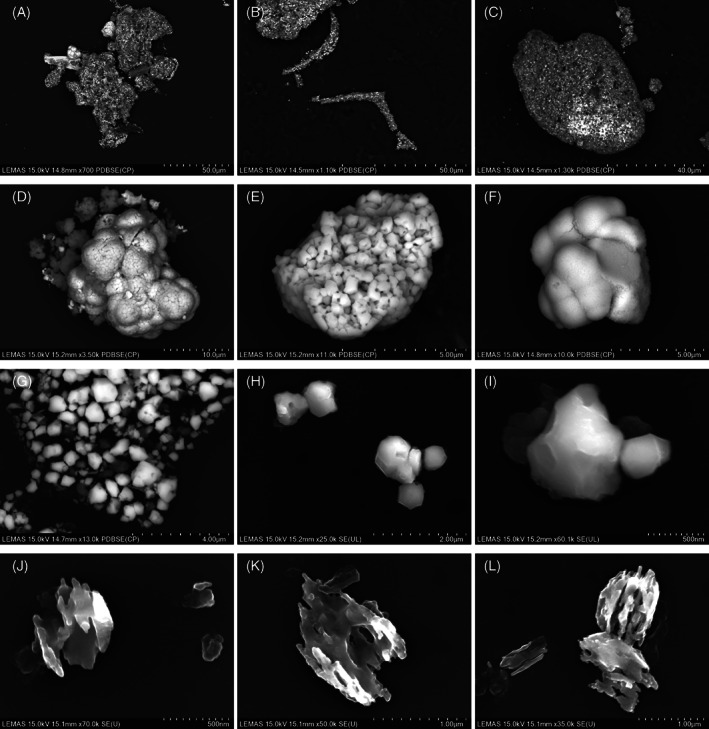
Examples of particles of bone cement. (A–C) Polymethylmethacrylate; (D–F) Mulberry‐like zirconium dioxide (ZrO_2_); (G–I) Granular ZrO_2_; (j‐l) Barium Sulphate

**FIGURE 4:**
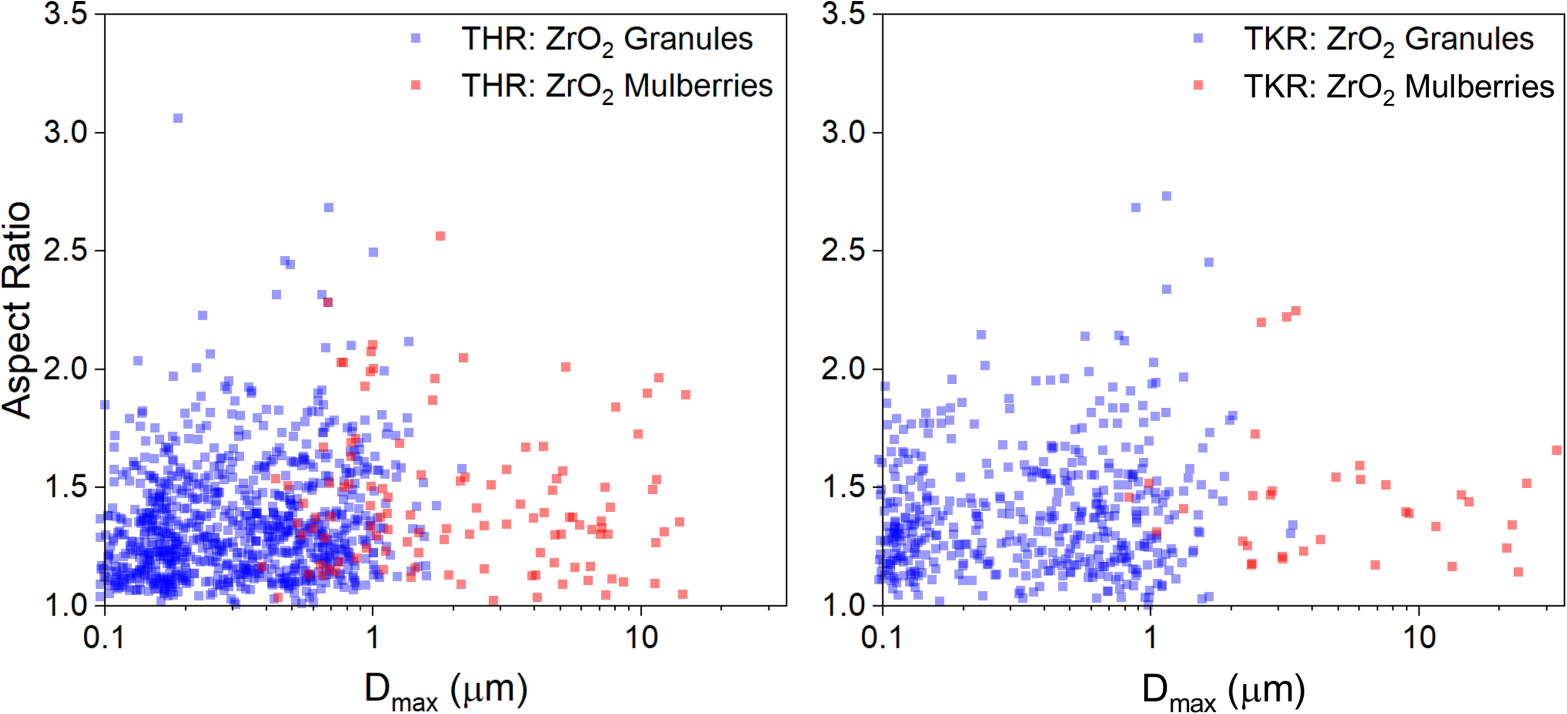
Size and aspect ratio of two types of isolated zirconium dioxide (ZrO_2_) wear particles in both total hip replacement (left) and total knee replacement (right) tissue samples. *D*_max_, major diameter. The abscissa is a logarithmic scale

PMMA particles were observed as relatively large (>10 μm), granular, irregular, angular morphologies but sometimes granular, irregular, smooth, and porous (Figure 3A–C). ZrO_2_ particles were always clearly embedded within the PMMA matrix and these particles were only identified in samples where individual ZrO_2_ particles were also isolated. ZrO_2_ particles were the most abundant particle type characterized and appeared in two morphologies: (1) globular, cauliflower‐like formations which have previously been described as mulberry‐like agglomerates^40^ (Figure 3D–F), and (2) granular, irregular, angulated particles (Figure 3G–I). The mulberry‐like particles were typically in the submicron to micron size‐range and differed to the granular ZrO_2_ particles, which were found to be in the nanometer to submicron size‐range. This was consistent with the observation that mulberry‐like particles were composed of ZrO_2_ granules. The granular composition of the mulberry‐like particles could be clearly identified at higher magnifications. Both ZrO_2_ particle types were similar in size and shape between THR and TKR samples (Figure 4). BaSO_4_ particles were only identified in two samples (1 THR and 1 TKR) but were easily identifiable due to their unusual appearance. These particles were granular, irregular and porous but atypical of the examples given in ASTM F1877‐16^38^ (Figure 3J–L).

**FIGURE 5:**
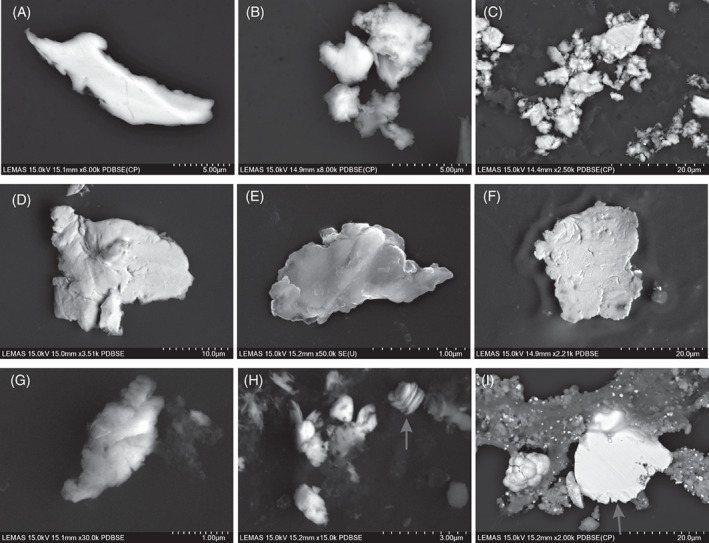
Examples of metallic particle types. (A–C) Titanium Alloy; (D–F) Stainless steel; (G–I) Cobalt chromium alloy (pink arrow identifies the specific cobalt chromium alloy particle)

Titanium alloy particles were the most frequently identified metallic wear particle type. These were typically flake‐like shards, in the micron size range with an elongated morphology (Figure 5A). However, titanium alloy also presented as flake‐like irregular particles (Figure 5B,C). Stainless steel wear particles were smooth and flake‐like, which were generally similar in size and morphology to titanium alloy particles, except generally larger and less elongated (Figure 5D–F). Cobalt chromium alloy was the rarest particle type with only 12 particles characterized from three samples (Figure 5G–I). These particles were the only type to manifest as flake‐like stacked‐sheets (Figure 5H).

**FIGURE 6:**
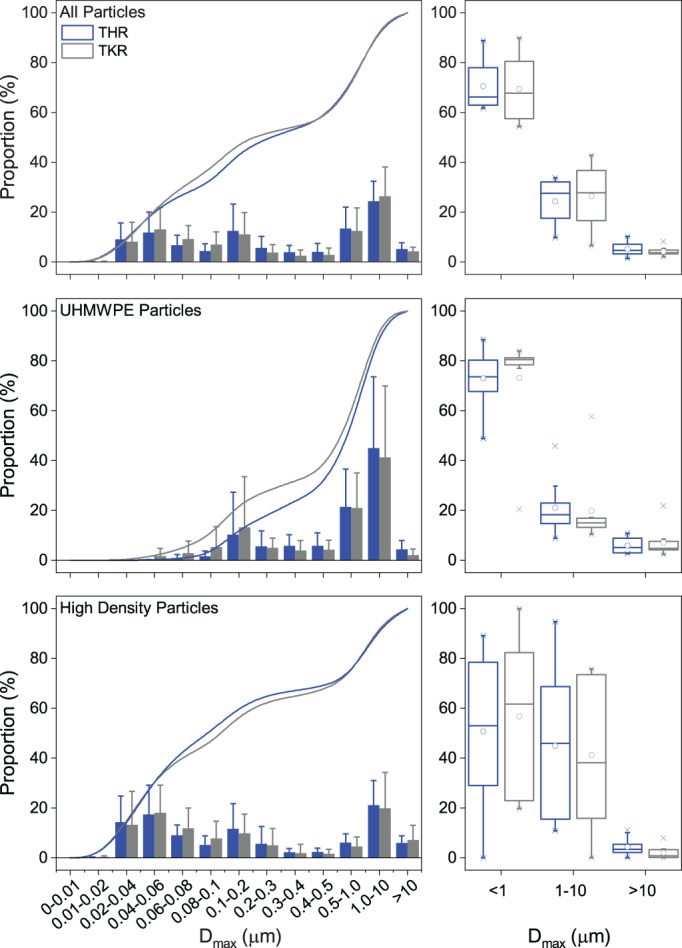
Wear particle size characteristics by joint type, material type and size range. Left sided graphs are the cumulative frequency of particles in each size range and the right sided graphs show the proportion of particles in each conventional size range. THR, Total hip replacement (blue); TKR, Total knee replacement (gray); UHMWPE, Ultra‐high molecular weight polyethylene; *D*_max_, major diameter

The proportion of particles observed in the three pre‐defined size ranges (<1, 1–10, and >10 μm) were similar between THR and TKR samples when all particle types were considered (Figure 6). However, there were notable differences between size ranges when comparing all low‐density particles combined to all high‐density particles combined. For example, 73% of all UHMWPE particles were in the submicron size range for THRs, whereas 51% of high‐density particles were submicron in size. Of all the wear particle types isolated, UHMWPE and ZrO_2_ particles were the only types to be predominantly in the submicron size range. All other material types were larger than 1 μm, on average (Table 3). PMMA was the only particle type to be consistently in the >10 μm size range. Relative particle size between material types were generally consistent and comparable between both joint replacement types.

**TABLE 3:** Characteristics of particles isolated from total hip and knee replacement

	Total hip replacement			Total knee replacement		
	*D*_max_ (μm) (median)	Aspect ratio (mean ± SD)	Roundness (mean ± SD)	*D*_max_ (μm) (median)	Aspect ratio (mean ± SD)	Roundness (mean ± SD)
All particles	0.25	1.54 ± 0.65	0.71 ± 0.16	0.34	1.73 ± 1.02	0.68 ± 0.20
Range	0.02–464.56	1.01–13.18	0.08–0.99	0.02–230.61	1.00–11.17	0.09–1.00
UHMWPE	0.09	1.52 ± 0.60	0.71 ± 0.16	0.08	1.50 ± 0.58	0.73 ± 0.16
Range	0.02–464.56	1.01–13.18	0.08–0.99	0.02–230.61	1.01–9.43	0.11–1.00
All high density	0.56	1.57 ± 0.72	0.71 ± 0.17	0.86	2.01 ± 1.33	0.61 ± 0.23
Range	0.06–65.37	1.01–10.72	0.09–0.99	0.04–76.97	1.00–11.17	0.09–1.00
PMMA	14.50	1.98 ± 0.86	0.60 ± 0.18	17.19	1.61 ± 0.30	0.63 ± 0.14
Range	5.03–65.37	1.05–3.93	0.25–0.95	6.05–76.97	1.13–2.06	0.49–0.89
All ZrO_2_	0.34	1.36 ± 0.26	0.76 ± 0.12	0.41	1.37 ± 0.25	0.74 ± 0.12
Range	0.06–14.69	1.01–3.06	0.33–0.99	0.04–32.84	1.00–2.73	0.37–1.00
ZrO_2_ mulberries	1.91	1.45 ± 0.31	0.72 ± 0.13	3.72	1.42 ± 0.25	0.71 ± 0.11
Range	0.38–14.69	1.02–2.56	0.39–0.98	0.83–32.84	1.14–2.25	0.45–0.87
ZrO_2_ granules	0.29	1.35 ± 0.25	0.76 ± 0.12	0.23	1.37 ± 0.25	0.75 ± 0.12
Range	0.06–2.15	1.01–3.06	0.33–0.99	0.04–3.39	1.00–2.73	0.37–1.00
BaSO_4_	1.12	1.51 ± 0.34	0.69 ± 0.13	0.72	2.11 ± 1.48	0.59 ± 0.20
Range	0.25–3.06	1.02–3.00	0.33–0.98	0.09–1.80	1.14–8.74	0.11–0.88
Ti alloy	1.05	2.14 ± 1.24	0.57 ± 0.22	1.72	2.91 ± 1.70	0.46 ± 0.22
Range	0.09–12.57	1.05–10.72	0.09–0.95	0.15–24.67	1.03–11.17	0.09–0.97
SS	3.13	1.76 ± 0.61	0.63 ± 0.17	2.83	1.68 ± 0.75	0.68 ± 0.22
Range	0.32–18.47	1.02–3.95	0.25–0.98	0.27–21.00	1.01–4.12	0.24–0.99
CoCr	8.61	3.00 ± 1.70	0.49 ± 0.28	5.89	2.20 ± 0.73	0.50 ± 0.16
Range	3.44–13.77	1.30–4.70	0.21–0.77	1.13–9.95	1.29–3.85	0.26–0.78

Abbreviations: *D*_max_, major diameter; SD, standard deviation; UHMWPE, ultra‐high molecular weight polyethylene; PMMA, polymethylmethacrylate; SS, stainless steel; CoCr, cobalt chromium alloy.

## 4 DISCUSSION

A modified wear particle isolation method was developed and implemented with the aim of isolating mixed wear particle populations of commonly used materials in TJRs from the same periprosthetic tissue sample. UHMWPE and a range of high‐density wear particle types were isolated from all tissue samples in this study, which confirmed the feasibility of the method. The isolation of several particle types from all the tissue samples processed in this research confirmed the presence of mixed particle populations, the majority of which were in the submicron size range. Approximately half of the tissue samples analyzed in this study were from devices revised for aseptic loosening after more than 10 years of implantation. How these particles influenced the biological response cannot be determined from this study, but recent research has linked such particle types to osteocytic osteolysis,^6^ which may be further aggravated by the presence of mixed particle populations. This study demonstrates the complex particulate environment challenging periprosthetic tissues at the bone‐implant interface of joint replacements.

Low‐density wear particles with a morphology and chemical composition typical of UHMWPE wear particles were identified in all THR and TKR tissue samples. All explants featured a UHMWPE bearing, which is generally considered to be the least wear‐resistant material within these constructs, therefore the presence of UHMWPE wear particles was expected, particularly given the long implantation times recorded for the majority of explants. The median UHMWPE wear particle size was 0.09 μm (IQR = 0.05–0.90) for THR and 0.08 μm (IQR = 0.05–0.64) for TKR. The majority of UHMWPE particles produced by THR and TKRs have previously been reported to be within the submicron size range (~0.1–0.8 μm),^25^, ^26^, ^34^, ^36^, ^41^, ^42^, ^43^ but nanometer‐sized granules^36^ and millimeter‐sized fibrils are also commonly identified.^33^ The broad spectrum of UHMWPE particle sizes and morphologies reflects the different wear processes, such as adhesion, abrasion or fatigue, occurring at the interfacing surfaces.^44^ Topolovec, et al.^37^ more recently noted that 51% of their isolated UHMWPE particles were smaller than 0.1 μm and that only 7% of particles were larger than 0.5 μm. The median UHMWPE particle size identified in this current study were slightly smaller than the mean values reported in the literature, which was expected as wear particle distributions are non‐parametric.^45^ The interquartile ranges for both joint types were consistent with the typical submicron UHMWPE size distribution reported in the published literature.

High density wear particles were isolated from all THR and TKR tissue samples in this study. The most common high‐density wear particle type identified was ZrO_2_, which presented as both mulberry‐like particles and granules for both joint types. In several samples, vast numbers of these particles were found embedded within larger PMMA particles isolated from the same tissue samples. Zirconium dioxide particles are an additive used in bone cement (PMMA) to act as a radiopacifier in a similar way to how barium sulphate is used. Manufacturers tend to add one or the other to bone cement to improve its visibility on X‐ray imaging. Bone cement particles have been isolated from tissue samples previously.^46^, ^47^, ^48^ Lerouge, et al.^49^ was the first and only study to isolate zirconia particles from tissue samples and found the average size to be 0.28 ± 0.08 μm. This was the same average ZrO_2_ granule size identified in the current study. Lerouge et al.^49^ did not report the presence of mulberry‐like agglomerates. Bos and Johannisson^48^ identified zirconium oxide and PMMA particles in the lymph nodes of 17 patients with THR using histology. These particles were typically between 0.02 and 0.75 μm in size, and both large mulberry‐like aggregates (up to 7 μm) and small granular particles of zirconium oxide were characterized. The larger PMMA particles were sized between 0.5 and 20 μm. These particle types and sizes were consistent with the findings of this current study and a recent study on the retrieval of bone cement.^40^ Bone cement related particles were found in 12 of the 14 tissue samples from explants confirmed to have at least one cemented component. The high prevalence of this particle type in the isolated tissue is likely due to the proximity of bone cement to the tissue and the matrix (PMMA) being less wear‐resistant compared to titanium alloy, cobalt chromium alloy and stainless steel. The presence of these ultra‐hard ZrO_2_ granules as third bodies within the joint space may also indicate a potential for accelerated wear of the cup/liner at the bearing interface, however the effect of this could not be determined by this study.

The majority of previous research into metal wear debris is focused on cobalt chromium alloy metal‐on‐metal hip replacements,^31^, ^45^, ^50^, ^51^ which tend to produce nanometer‐sized (~30–100 nm) metallo‐organic composite spheroids.^44^ However, metal wear particles are also commonly found within periprosthetic tissue surrounding non‐metal‐on‐metal THR and TKR,^19^, ^37^, ^52^, ^53^ which is more relevant to the devices in this study. The composition, size and shape of such metal particles depends on the source, location and the mechanism of wear. For example, trunnion wear against the inside of a ceramic THR head has been shown to produce titanium alloy wear particles sized between 0.02 and 0.05 μm,^54^ whereas titanium alloy wear debris produced at the stem, acetabular cup or supporting screws of ceramic‐on‐ceramic THRs were substantially larger at 0.61 ± 0.31 μm.^49^ The titanium alloy wear debris isolated in this current study was predominantly micron‐sized and elongated. The origins of the titanium alloy wear particles in the THR samples are the cup or stem components. One explanted TKR had a worn‐through tibial tray which resulted in a large number of titanium alloy particles being isolated from the associated tissue sample. For the other TKR samples, less obvious wear of the tibial tray was the likely origin of the particles.

Only a very small number of cobalt chromium alloy and stainless steel wear particles were characterized in this current study. The tissue samples were predominantly from surrounding cemented devices and the layer of bone cement separating the tissue from the metallic components could have reduced the probability of exposure to such particles. Cobalt chromium alloy particles were found predominantly in one TKR tissue, which featured cobalt chromium alloy femoral and tibial components. The flake‐like stacked sheet morphology of the particles was indicative of abrasive wear, which is expected of TKRs.^19^ The origin of the stainless steel particles was unclear for several samples but the possible sources include monoblock hip stem components, which was stainless steel for three samples; accessory components such as a Posterior Lip Augmentation Device (PLAD),^55^ which was noted for one of THR systems included in this study; and/or the surgical instrumentation used at implantation/retrieval. Stainless steel scissors and scalpels were also used to dissect the tissue samples into small pieces in preparation for digestion and may have introduced contamination despite the significant care taken not to do so.

The method developed for this study implemented several techniques to capture the full particle size range, including ultra‐high resolution imaging, nano‐scale filtration pore sizes and manual characterization. Imaging magnification has been shown to be a critical factor for accurately characterizing particle size distributions.^56^ The earliest wear particle isolation studies were unable to confidently analyze nanometer wear particles due to the resolution of their analysis methods.^24^, ^26^ For example, the mean size of UHMWPE wear particles surrounding THRs were reported to be 0.53 ± 0.3 μm,^25^ and between 0.58 and 0.79 μm,^26^ both of which were determined using automated particle sizers. However, the maximum resolution of these automated methods was 0.4^24^, ^57^ and 0.58 μm,^26^ both of which are too low to precisely characterize the majority of particles produced in the nanoscale and sub‐micron size ranges. The resolution of these automated methods are approximately 20 times larger than the smallest particle identified in the current study. Tipper, et al.^58^ used manual characterization to observe nanometer wear particles using magnifications up to 65,000 times. Magnifications up to 200,000 times were used for wear particle characterization in the current study, which in part explains why the average particle size was smaller than the previous literature and is consistent with the prediction that improvements in technology result in smaller wear particle size distributions.^56^

The filter pore size used in this study was 0.015 μm which is smaller than other published methods that typically use 0.1 μm or larger.^59^ Using pore sizes too large for the expected particle types means nanometer‐sized particles can flow freely through the filter, resulting in their omission from the analysis. Nanometer wear particles have previously been isolated but as they commonly present in larger agglomerates, manual characterization may be more effective than automated methods at accurately identifying them.^36^ It is known that manual characterization is labour intensive, subject to observer selection bias and that fewer particles can be analyzed as a proportion of the total wear particle population.^36^ However, manual characterization was deemed necessary for the current study because the types of particles present in the tissue samples were unknown and required careful assessment to be accurately identified, imaged, and characterized.

This study tested the feasibility of a novel isolation method, which performed acceptably because UHMWPE and many of the high‐density wear particles associated with the patient's devices were isolated from all 18 tissue samples with minimal contamination. The processed tissue samples were however, from largely unknown failed THR and TKR devices and the type of UHMWPE used in these devices was also unknown. Differences between the material types (e.g., GUR 1020, GUR 1050, crosslinked etc.) would likely have added to the high variability of particle characteristics found between samples. Also, the anatomical location of where the sample was extracted from was not known and may also have affected the observed wear particle size distribution.^24^

EDX analysis is not sufficient by itself to determine the presence of UHMWPE wear particles because its carbon–oxygen composition is indistinguishable to the filter membrane and carbon coating used during characterization. However, in‐house research expertise, personal experience and EDX analysis were combined to visually identify particles with morphology similar to those identified in previous research. Contaminants such as atmospheric silica particles could be excluded based on their elemental composition. The characterization and verification by EDX analysis of individual particles could be viewed as a strength of using manual particle characterization and give confidence to the identification of the particles. However, given billions of particles are produced by each joint replacement, the relatively small sample of characterized particles may be subject to selection bias during manual characterization. A different wear particle size distribution may have resulted if automated analyses were used instead, however both approaches have their strengths and weaknesses.

The full breadth of the available magnification provided by the SEM was utilized in this study to identify and characterize individual particles. Because of this, more particles at the extremes of the size range would be expected to have been captured which, appears to have been reflected in the results. This differs from automated particle sizers which have a fixed size range and cannot discriminate between target particles and contaminants. Manual characterization also affected the number of particles characterized. Only approximately 100 to 400 UHMWPE wear particles were characterized per sample, the distribution of which may not be representative of the billions of wear particles produced by a joint replacement.^36^ Future research should focus on simplifying the method and implementing it in tissues samples from known joint replacement device combinations. A method with this capability may also be useful to further understand the heterogeneity of wear particle populations between sample selection sites.^53^

## 5 CONCLUSION

It has been 25 years since the first wear particle isolation methods were developed, over which time the simplicity and effectiveness of such methods have proved to be inadequate for processing of tissues surrounding modern bearing couples. This feasibility study demonstrates the ability to capture high resolution images of a range of different material types of isolated wear particles that are representative of the total wear particle burden within an individual tissue sample. This study identified the prevalence of ceramic wear particles within the tissues of patients with cemented total joint replacements and also the relative scarcity of cobalt chromium alloy wear particles in the same devices. The images and particle characteristics included in this study could provide a reference for researchers attempting to emulate clinically relevant wear particles for use in in vitro biological response studies.

## CONFLICT OF INTEREST

The authors declare no conflict of interest.

## Data Availability

The data supporting the findings of this study are available through the University of Leeds Respository^60^.
